# Masks: What They Can and Cannot Do

**Published:** 1919-03-15

**Authors:** 


### MASKS.

### What they Can and Cannot Do.

There are so many face appliances known by this name and used under medical circumstances that no doubt much of the recent argument concerning them has arisen by comparing results from widely different modifications. When the Local Government Board issued its influenza memorandum, it described, among many other things, the mask. The definition makes it appear as gauze (four layers) or butter muslin (three layers), which covers mouth, nose and chin, and its use is advised only when nursing or in attendance on a person suffering from the disease. There the official reference appears to end and with it much of the newspaper discussion as to their value, but, nevertheless, the position remains very far from being either explained or understood and is unsatisfactory, not only with regard to influenza, but also the whole question of germ transference and contagion in the numerous diseases whose causal organisms may be found in the oral and respiratory secretions.

### Protective Application.

The subject must be considered from two distinct standpoints. First, let us take the apparently uninfected person who covers the face with view to self-protection. In a recently published article by Leonard Hill, M.B., F.R.S., it is pointed out that the passage over the respiratory membranes of cool air, cool and therefore of low vapour tension, brings an increased flow of blood, increased evaporation, and therefore greater flow of tissue lymph through them. Experiments and observations showing the natural protective effects are quoted, and the author, in making comparison, states that "warm moist atmospheres are against this natural washing and immunising process." Also it is suggested that cool air, with cooling effect on the membranes, may tend directly to inhibit the growth of more delicate pathogenic bacteria. Now here is a direct reason why people should neither frequent nor live in crowded, warm, or ill-ventilated places and a simple proof of accepted maxims, but it has also a direct bearing on the question of face-covering. Even if the mask be frequently changed—and the memorandum recommends that it should be—the gauze is rapidly warmed and moistened by the out-going breath and inevitably results in the raising of both temperature and humidity of all inspired air before it reaches the mucous membranes. If it be assumed that, during an epidemic, the organisms are present in the respiratory passages of such apparently well people, we see the mask as aiding in the production of bacterial development, toxin absorption and febrile symptoms without any new germs coming from without. In our own experience any gauze covering becomes extremely moist in but a few minutes when any muscular exertion is undertaken and a moist mask may, then, even collect bacteria from the minute particles in the air and transfer them via the lips to the fauces.

Such reasons fall among many others incident to both health and comfort which justify the Board's advice against general protective wearing of masks. At the bedside, a dry clean gauze will screen a nurse from the results of sudden coughing, etc., in fact, catch all the larger particles of moisture, and therefore considerably reduce the infection risk, but it must be remembered that dry fabrics even in several layers are permeable to the very finest particles. By many authorities these are considered to be the chief means of transference, and if they are to be controlled by a mask, then theoretically it should be worn by the infected patient.

### Preventive Application.

Thus we reach our second standpoint; what is the value of a mouth and nose covering applied to the patient? As before, the warm moist air inspired in continuous use is detrimental, the discomfort to a sick man considerable, but to judge of the degree to which the dispersal of germs is prevented we must refer to the findings of Professor Sahli and other workers in Berne. Their experiments were made with gauze fabric masks and a plaster cast of the face. A nebulized watery suspension of B. prodigiosus was found to pass freely through the material at air speeds considerably less than that of normal respiration. In real life, though some of the coarser particles would be held back, yet in every case a large number of organisms must always escape into the air. Recent investigations suggest that, whatever its other characters, the causal agent of influenza is of very minute size, and judging from the growth of common bacteria produced twenty-four hours after allowing the breath to momentarily impinge upon a sterile culture plate. It seems clear that millions of germs from the patient would penetrate any mask of a thickness even remotely possible for the comfort of a case whose breathing is already difficult. Here again the advice of the memorandum appears sound as no really good purpose would be served.

A possible explanation of the one-time enthusiasm arises from the fact that, during the summer and autumn epidemics, there appeared to be a definite fall in case incidence when both the Americans and ourselves adopted the principle of distributing masks to troopships, hospitals, etc. It was at exactly the same time, however, that all realised the extent and menace of the epidemic. Medical examinations of men entering ships or camps were much more complete, isolation quicker, and in a thousand ways the mechanism of control developed. Nowadays authorities are practically unanimous in the view that general measures and never the mask brought about the improvement. The American troop convoys give a clear instance. Improved supervision on embarkation at once reduced the number of cases on board ship. Although masks were at the same time introduced and given a great deal of the credit,

**Masks**—(*continued*).

it was found on later voyages, when the disease was still active on both sides of the ocean, that the discarding of them, except for temporary wear by doctors and attendants, was followed by no increase in the number of cases.

On the whole the value for masks falls far short of popular belief. Undoubtedly they may act as a temporary and conveniently portable screen for those whose duties bring them into the close contact with infection and may, for such purpose, be elaborated to a greater efficiency than could ever be attained with simple gauze, but in everyday life, and quite apart from æsthetc considerations, there is ample justification for the authorities who suggest only their limited application.

